# In vivo characterization of rodent cyclic myocardial perfusion variation at rest and during adenosine-induced stress using cine-ASL cardiovascular magnetic resonance

**DOI:** 10.1186/1532-429X-16-18

**Published:** 2014-02-18

**Authors:** Thomas Troalen, Thibaut Capron, Monique Bernard, Frank Kober

**Affiliations:** 1 Aix-Marseille Université, CNRS, CRMBM UMR 7339, 27 Bd Jean Moulin, 13385 Marseille Cedex 5, France

**Keywords:** Myocardial blood flow, Microcirculation, Adenosine, Perfusion, Rat heart

## Abstract

**Background:**

Assessment of cyclic myocardial blood flow (MBF) variations can be an interesting addition to the characterization of microvascular function and its alterations. To date, totally non-invasive in vivo methods with this capability are still lacking. As an original technique, a cine arterial spin labeling (ASL) cardiovascular magnetic resonance approach is demonstrated to be able to produce dynamic MBF maps across the cardiac cycle in rats.

**Method:**

High-resolution MBF maps in left ventricular myocardium were computed from steady-state perfusion-dependent gradient-echo cine images produced by the cine-ASL sequence. Cyclic changes of MBF over the entire cardiac cycle in seven normal rats were analyzed quantitatively every 6ms at rest and during adenosine-induced stress.

**Results:**

The study showed a significant MBF increase from end-systole (ES) to end-diastole (ED) in both physiological states. Mean MBF over the cardiac cycle within the group was 5.5 ± 0.6 mL g^-1^ min^-1^ at rest (MBF_Min_ = 4.7 ± 0.8 at ES and MBF_Max_ = 6.5 ± 0.6 mL g^-1^ min^-1^ at ED, *P* = 0.0007). Mean MBF during adenosine-induced stress was 12.8 ± 0.7mL g^-1^ min^-1^ (MBF_Min_ = 11.7±1.0 at ES and MBF_Max_ = 14.2 ± 0.7 mL g^-1^ min^-1^ at ED, *P* = 0.0007). MBF percentage relative variations were significantly different with 27.2 ± 9.3% at rest and 17.8 ± 7.1% during adenosine stress (*P* = 0.014). The dynamic analysis also showed a time shift of peak MBF within the cardiac cycle during stress.

**Conclusion:**

The cyclic change of myocardial perfusion was examined by mapping MBF with a steady-pulsed ASL approach. Dynamic MBF maps were obtained with high spatial and temporal resolution (6ms) demonstrating the feasibility of non-invasively mapping cyclic myocardial perfusion variation at rest and during adenosine stress. In a pathological context, detailed assessment of coronary responses to infused vasodilators may give valuable complementary information on microvascular functional defects in disease models.

## Background

The pulsatility of coronary flow has been studied for several decades [1,2]: as elsewhere in the arterial circulation, it is the pressure difference between the aorta and the peripheral capillaries that provides the driving force for coronary circulation. In a paradoxical way, the heart, which is dedicated to supply blood to the organs, restricts its own blood supply during peak contraction in systole unlike most other systemic vascular beds which have a fresh oxygenated blood supply in systole. The squeezing effect of myocardial contraction causes arterial blood inflow to peak during diastole and venous blood outflow to peak during systole [3,4]. Microvessels are abundant in the myocardium so that myocardial blood volume (MBV) and flow (MBF) vary during the cardiac cycle in response to this pulsatile change in coronary circulation and cyclic variation in myocardial tension. Diseases such as diabetes, atherosclerosis, cardiomyopathies, and arterial hypertension result in functional and morphologic microvascular alterations, which may precede clinical signs and symptoms. The mechanical interaction between coronary microcirculation, and myocardial contraction and relaxation is fundamentally important for an understanding of intramural coronary hemodynamics. The morphology and function of capillaries across myocardial wall during a cardiac cycle play a pivotal role on the mechanical control of blood perfusion to regional myocardium together with arteriolar and venular mechanics and responses [5]. Determination of the role of systolic extravascular compression on the pattern of blood flow in the microcirculation during the cardiac cycle has been limited to measurements made in those regions of the myocardium where direct observations of these microvessels is possible [6-8]. Quantitative assessment of myocardial perfusion and characterization of cyclic myocardial perfusion variation would help better understand the mechanisms underlying myocardial contractility and may give valuable complementary information on microvascular functional defects in non-ischemic heart diseases.

Cyclic change of regional MBV in the mouse heart has already been reported using cardiovascular magnetic resonance (CMR) based on the correlation with cyclic variation in the steady-state *T*_2_^*^ shortening effect of an ultrasmall iron oxide nanoparticle intravascular contrast agent [9]. Another study focused on noninvasive MBV variation assessment in the canine left ventricle using displacement encoding with stimulated echoes (DENSE) technique [10]. Nevertheless, no study has assessed MBF variation over the entire cardiac cycle in the rodent heart. In humans, however, Radjenovic et al. demonstrated the feasibility of assessing systolic and diastolic MBF changes by dynamic contrast-enhanced CMR [11].

Even if first-pass CMR has been proved to quantitatively assess MBF [12-15], arterial spin labeling (ASL) has become a method of choice to quantitatively and non-invasively map rodent MBF [16-24]. ASL has also shown its potential to quantify human MBF [25,26]. Cine-ASL has recently been proposed as an original approach to assess MBF [27,28]. This technique is based on a combination of a continuous cine CMR gradient echo readout with a steady-pulsed arterial labeling approach. This scheme provides quasi-continuous tagging of the blood feeding the coronaries while maintaining compatibility with the constraints of cardiac motion and the highly pulsatile blood flow in the ascending aorta. The steady-state approach associated with the cine readout allowed us to dynamically map MBF over the entire cardiac cycle with high temporal resolution. In a preliminary study, cyclic change of regional MBF in healthy mice was measured at rest [29], and a significant MBF increase of 30% from end-systole (ES) to end-diastole (ED) was shown. Based on this finding, we propose a new protocol which compares cyclic MBF changes in healthy Wistar rats between rest and adenosine-induced stress.

## Methods

### Animal preparation

All experiments were conducted according to a protocol approved by the University’s animal experimentation committee. Seven healthy female Wistar rats (age 25 weeks, body weight 329 ± 8 g; Janvier Laboratories, Le Genest-Saint-Isle, France) were anesthetized in an induction chamber with 3% isoflurane. During the experiments, a 2% dose of isoflurane mixed with 1L min^-1^ of pure oxygen gas stream was continuously delivered through a face mask. For the present study, isoflurane was chosen as it maintains cardiac function and permits real time regulation of the depth of anesthesia whereas barbiturates such as pentobarbital are known to have depressant effects on hemodynamic parameters [30]. Nonetheless, isoflurane has been shown to induce coronary vasodilation and MBF increase at rest compared to other anesthetics [31]. Isoflurane concentration was regulated using a dedicated vaporizer (Ohmeda/General Electric, Milwaukee, WI, USA) so as to obtain regular breathing frequencies in the range of 70 breaths per minute. Respiration was monitored using a pressure sensor connected to an air-filled balloon positioned on the back of the rat. Body temperature was monitored using a rectal probe and maintained at 37°C using a heating blanket with hot water circulation placed around the rat. The electrocardiogram (ECG) signal was monitored by placing two subcutaneous electrodes, one in the right foreleg and one at abdominal level. The electrodes were connected to an ECG trigger unit (Rapid Biomedical, Rimpar, Germany) to record the signal and to trigger the CMR sequence. Oxygen saturation was also monitored to ensure stable physiological conditions along the experiment. A catheter was placed in the tail vein to deliver adenosine. The venous cannula had a dead volume of about 120 μL.

### Perfusion CMR

Cine-ASL has recently been proposed as an original ASL scheme to assess MBF in small animals. Theory of the technique and validation against another ASL approach are described elsewhere [27,28]. Briefly, cine-ASL relies on a fast ECG-gated cine Fast Low Angle Shot (cine-FLASH) sequence repeated over several cardiac cycles for each line of k-space. At each cardiac cycle, one single gradient-echo is substituted by a spatially selective inversion pulse, labeling the arterial blood at the level of the aortic root. This labeling has to be performed just before the arterial blood enters through the myocardial tissue via the coronaries, i.e. at ES. Using this approach, a continuous cine acquisition can be performed while the blood magnetization feeding the coronaries is driven into a steady-state, leading to a perfusion-dependent stationary regime of tissue magnetization. The repetition time TR between each gradient echo was kept as short as possible to maximize temporal resolution, six milliseconds in these experiments.

**Figure 1:**
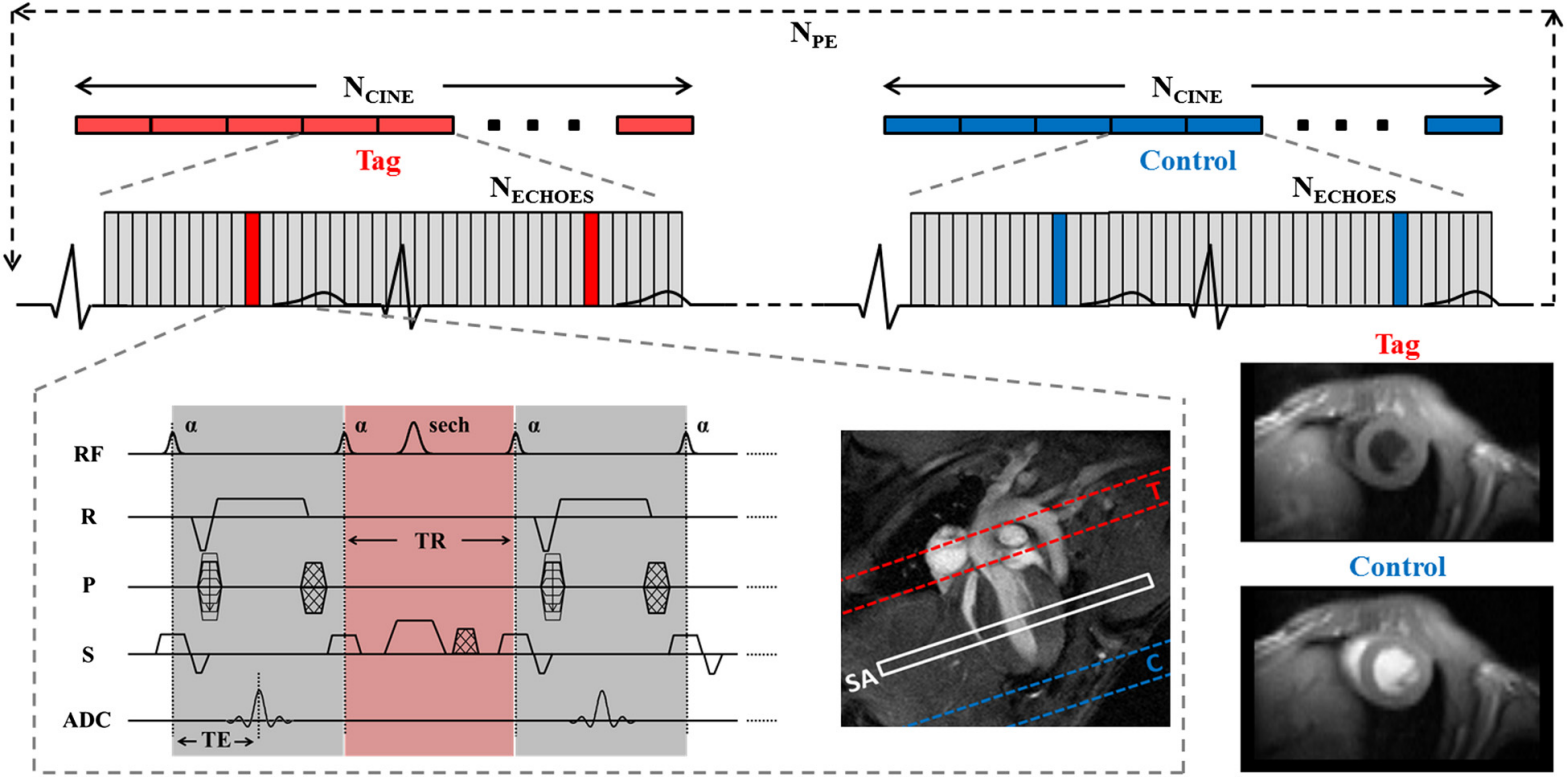
**Schematic description of the cine-ASL pulse sequence.** The pulse sequence gated on the ECG signal (top) and the corresponding chronogram of the modified cine-FLASH are shown (bottom left). Slab positions are displayed on a four-chamber long-axis view and resulting tag and control images are displayed (bottom right). The sequence parameters are N_echoes_ (number of echoes), N_cine_ (number of cine blocks), N_PE_ (number of phase encoding steps).

As for all prospectively gated techniques, the original sequence had to be stopped before the next ECG trigger so that the observable time window within the cardiac cycle was only about two third of the cardiac cycle. Also, the waiting delay caused steady-state interruptions that might bias the perfusion signal on the short time scale observed here. Compared with the original cine-ASL version [27], the sequence was therefore slightly modified so as to acquire during almost two cardiac cycles (Figure 1) after each trigger pulse. We assumed that the heart rate was constant over two cardiac cycles so that ECG-triggering every two heart beats was sufficient. By appropriately adjusting the number of cardiac phases to acquire per cardiac cycle as well as the number of echoes at which the labeling pulses occur, more than one cardiac cycle coverage without interrupting the steady-pulsed labeling scheme was possible. In summary, this modification was done for two reasons: (i) the cine readout covered almost two cardiac cycles with a negligible interruption of the FLASH steady-state, and (ii) the labeling was still performed every heart beat which is mandatory to optimize labeling efficiency (see perfusion quantification section below).

### Experiment protocol

All imaging experiments were performed on a Bruker Biospec Avance 4.7T/30 imager with horizontal bore (Bruker, Ettlingen Germany). Homogeneous RF excitation was achieved using a proton volume resonator (diameter 60 mm, homogeneous length 80 mm), and the animals were positioned prone on an actively decoupled surface receive coil (Rapid Biomedical, Wurzburg, Germany). Prior to the perfusion measurements, scout images were acquired to determine the short-axis plane used for the perfusion imaging sequences. A cine-FLASH sequence in four-chamber long-axis view was used to achieve precise spatial and temporal placement of the labeling pulses at ES, just before aortic valve closure.

Perfusion measurements were carried out in the short-axis plane using cine-ASL with the following parameters: imaging slice thickness: 2 mm, field of view: 40 mm, in-plane resolution: 312 × 625 μm^2^, TE/TR: 1.64/6 ms, flip angle α: 8° (gaussian pulses, 0.5 ms), labeling slice thickness: 6 mm (adiabatic hyperbolic secant pulses, 3.7 ms). The number of acquired cardiac phases (N_echoes_) was adjusted according to the animal’s heart rate, about 30 echoes per cardiac cycle and 50 echoes per cine block. Acquisition time was approximately 12 min at 400 bpm. All animals first underwent MBF quantification at rest. Continuous adenosine infusion was accomplished using a dedicated low-flow pump injector (World Precision Instruments, Sarasota, FL, USA) and a tail vein catheter. The infusion rate was set to 280 μg kg^-1^ min^-1^, which corresponds to twice the standard clinical rate used in human clinical practice. This rate was shown in preliminary experiments to reliably ensure a maximal response to adenosine. The second cine-ASL sequence was started 5 minutes after an initial heart rate drop observed upon arrival of adenosine and infusion was maintained until completion of the sequence. The criterion for appreciation of a hyperemic state was a significant increase in MBF compared to rest condition.

### Perfusion quantification

Global MBF maps in left ventricular myocardium were computed from the steady-state perfusion-dependent gradient-echo cine images. As a result of the theory presented earlier [28], the experimentally measured relevant quantity is the stationary signal difference between control and tag scans $\Delta M_{\infty }=M_{\infty }^{c}-M_{\infty }^{t}$, which is related to myocardial blood flow by the following equation:

$$\mathrm{MBF}=\frac{\lambda M_{\mathrm{SS}}}{T_{1}^{*}M_{0}}\frac{\frac{\Delta M_{\infty }}{M_{\infty }^{c}}}{2\beta -\frac{\Delta M_{\infty }}{M_{\infty }^{c}}}$$

where, *β* corresponds to the average labeling efficiency (*β* = 1 for a complete inversion and *β* = 0.5 for a saturation), λ = 0.95 the blood-tissue partition coefficient for water [32] and $T_{1}^{*}$ the apparent relaxation time measured under the influence of rapid succession of small flip angle pulses [33]. *T*_1_ was fixed to 1.31 s as reported in a previous study [18]. M_SS_ is the longitudinal steady-state magnetization observed under FLASH partial saturation and can be directly calculated from *M*_0_, TR, *T*_1_ and the excitation flip angle α:

MSS=M01−e−TRT11−cosα.e−TRT1

As reported in previous work [27], the labeling efficiency reached with this particular steady-pulsed scheme was only 0.5, although adiabatic inversion pulses were used. Considering the large extent of the inversion slice of 6mm which covers both atria, pulmonary compartments and the aorta, blood entering into the coronaries has already been inverted several times with different inversion delays before reaching the microvascular compartment. It has been shown that this multiple exposure of blood spins to inversion pulses led to reduced labeling efficiency *β* of 0.5, confirmed by absence of signal in the LV blood pool as also observed earlier [27].

### Image processing

To perform image analysis, an in-house developed program running in an IDL environment (ITT, Boulder, CO, USA) was used, and perfusion maps were generated by applying this model pixel-by-pixel. Transmural MBF was quantified as an average of pixel values for regions of interest drawn on every map in the series (about 50 maps per acquisition). Global myocardium was first evaluated from the MBF maps for each rat as a function of time. Since the blood signal coming from the left ventricle and from the coronaries was significantly higher than the measured signal of interest, particular attention was paid at masking this signal on each individual map. Indeed, coronary flow is known to be highly variable within the cardiac cycle [34], and taking blood signal into account could lead to a misinterpretation of cyclic myocardial perfusion variation.

Since the cyclic variations observed at rest and during stress were dependent on the animal’s heart rate, data were temporally normalized with respect to the individual RR interval. In order to perform a group analysis, MBF data were sorted into bins representing the mean temporal resolution after the normalization process. Finally, myocardial perfusion reserve (MPR) was computed as a ratio of stress and rest MBF for each temporal bin.

To obtain the average change of MBF over the cardiac cycle, the two highest and lowest values found at end-diastole (ED) and end-systole (ES), respectively, were averaged so as to calculate the relative cyclic variation defined as ΔMBF_Cycl_ = (MBF_Max_ - MBF_Min_)/MBF_Max_. Measurements of systolic and diastolic MBF were done in the first cardiac cycle for each individual rat at rest and during stress before averaging within the group. To assess inter-observer variability, all measurements were performed twice by two different readers.

### Statistical method

Data were presented either as group mean value ± SD across the entire cardiac cycle or as group mean value ± SD for each temporal bin within the cardiac cycle. All statistical processing was performed using Prism 5 software (Graph Pad, San Diego, CA, USA). Statistical differences between systolic and diastolic values of MBF and MPR, i.e. minimum and maximum, were evaluated using a paired *t*-test. A probability value of <0.05 was taken as statistically significant. Correlation between heart rate and relative cyclic variation ΔMBF_Cycl_ was quantified by the Pearson rank correlation coefficient rho. A paired *t*-test was also used to compare cyclic variation measurements between the two observers.

## Results

**Figure 2:**
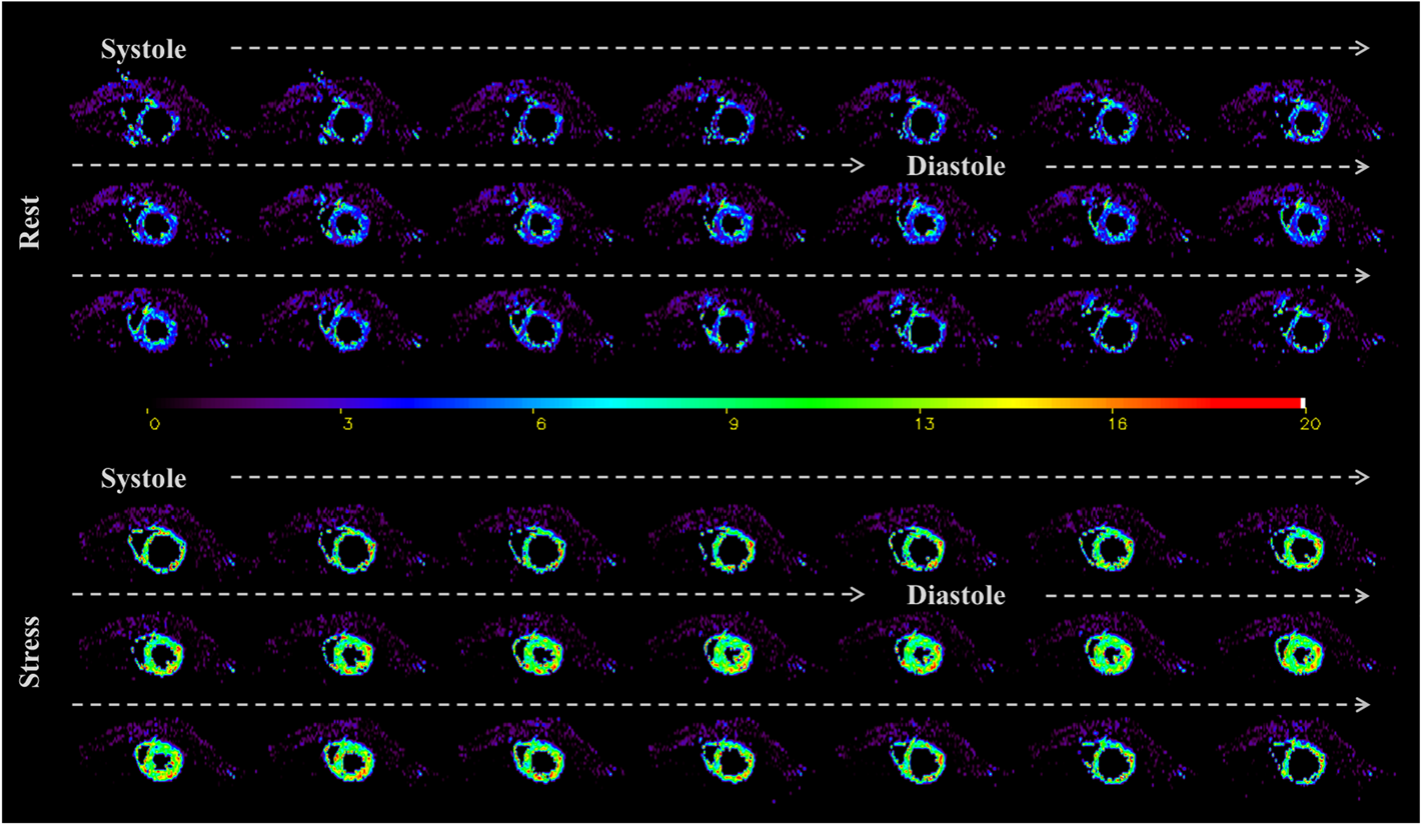
**Dynamic MBF mapping.** Twenty one consecutive color-coded short-axis perfusion (MBF) maps resulting from a measurement series over the cardiac cycle. The color scale is in mL g^-1^ min^-1^. Data are shown from the same normal rat heart at rest (top) and during stress (bottom) while 280 μg kg^-1^ min^-1^ of adenosine were administered intravenously inside the magnet. Images were acquired using the cine-ASL sequence with in-plane resolution and slice thickness of 312 × 625 μm^2^ and 2 mm, respectively.

The Cine-ASL approach allowed for dynamic quantification of MBF across the cardiac cycle with time-frames given by the cine repetition time of 6 milliseconds. Figure 2 shows an example of a perfusion map series over the entire cardiac cycle obtained with cine-ASL from the same rat at rest and during adenosine stress. By inducing strong hyperemia with perfusion significantly increased by a factor of 2.1. In this example, mean perfusion over the entire cardiac cycle in the global myocardium increased from 6.3 ± 0.6 mL g^-1^ min^-1^ at rest to 13.7 ± 1.3 mL g^-1^ min^-1^ during stress. This result demonstrates that cine-ASL has the potential to dynamically map MBF over the cardiac cycle and to study and quantify changes in myocardial perfusion within the cardiac cycle for different physiological states. In terms of breath and heart rates, no statistical differences were found between experiments at rest and during stress (Table 1).

**Table 1:** Physiological parameters recorded during the experiment

**Rat**	**Body weight (g)**	**Heart rate (bpm)**		**Breath rate (bpm)**	
		**Rest**	**Stress**	**Rest**	**Stress**
1	320	370 (±30)	387 (±40)	65	70
2	330	359 (±10)	341 (±20)	70	80
3	325	261 (±40)	256 (±50)	60	65
4	338	359 (±15)	308 (±20)	65	60
5	337	286 (±10)	300 (±20)	60	65
6	320	310 (±5)	330 (±30)	75	70
7	335	308 (±10)	323 (±20)	65	60
Mean	329	322	321 ^ns^	68	67^ns^
SD	8	42	40	7	8

No statistical differences (ns) were found between rest and stress experiments.

### Capillary flow dynamics

**Figure 3:**
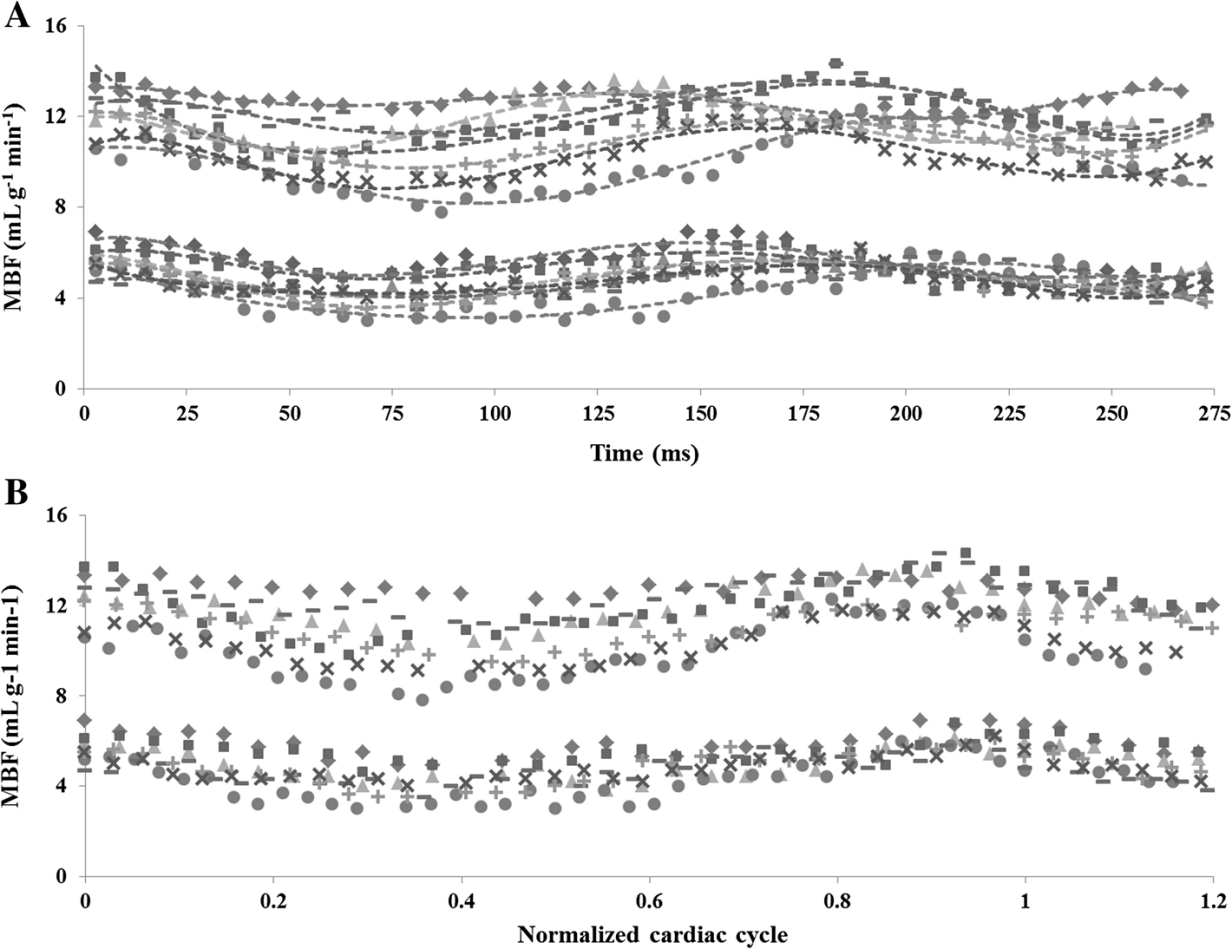
**Temporal normalization process.** All individual cyclic perfusion dynamics **(A)** were rebinned relatively to heart rate resulting in curves that represent the MBF evolution as a function of the normalized cardiac cycle **(B)**. Once the data are normalized, a general behavior emerges along the cardiac cycle with variation within the group depending on individual response to adenosine.

**Figure 4:**
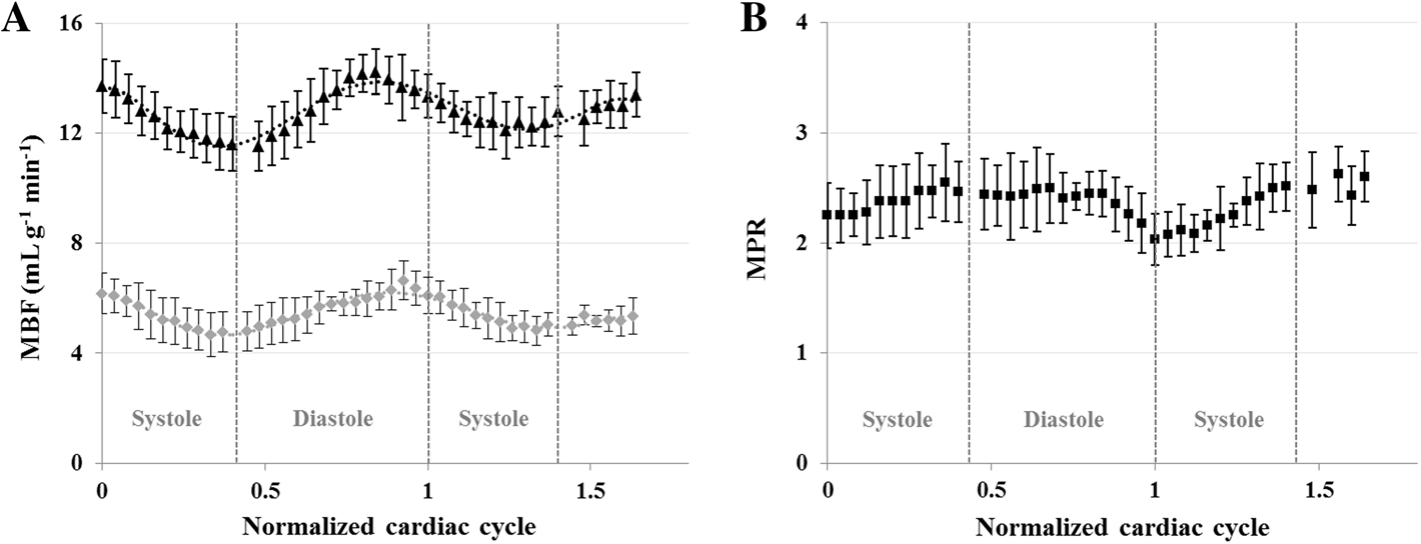
**Cyclic myocardial perfusion variation over the cardiac cycle. A)** Mean MBF within the group of seven healthy rats are reported as a function of the normalized cardiac cycle. These are shown at rest (grey diamonds) and during adenosine-induced stress (black triangles). For clarity, a six orders polynomial regression was used to fit the data. Error bars correspond to the standard deviation within the group for each temporal bin. Minimum MBF was found to be at ES for both physiological states. Maximum MBF was found to be at ED at rest and sooner than ED during stress. **B)** MPR values are given as free ratios of stress and rest MBF. MPR behavior (black squares) was found reversed compared to MBF variation, with first an initial increase during systole until a maximum at ES. This maximum value was conserved during most of diastole with a minimum at ED.

In Figure 3A all individual MBF data sets observed in the seven normal rats studied are shown as a function of time, at rest and during adenosine stress. Figure 3B shows normalized data over the cardiac cycle. Figure 4A shows the evolution of MBF across the cardiac cycle at rest and during adenosine-induced stress. These results are plotted as a function of the normalized cardiac cycle averaged over the seven animals studied ± standard deviation within the group. Along the series starting at early-systole, perfusion gradually and significantly decreased, reaching a minimum at ES and then recovered during the diastole. This general behavior was well reproducible in all subjects. The maximal perfusion values were always found to beat ED just before the next QRS complex. One can also notice a slight shift of the maximum MBF during stress compared to rest which was found to be reached sooner within the cardiac cycle. In Figure 4B, averaged MPR within the group is reported as a function of the normalized cardiac cycle. MPR behavior was found reversed compared to MBF variation, with an initial increase during systole until a maximum at ES. This maximum value was sustained during most of the diastole. Towards the end of the cardiac cycle, MPR dropped in a sudden way reaching a minimum at ED.

**Figure 5:**
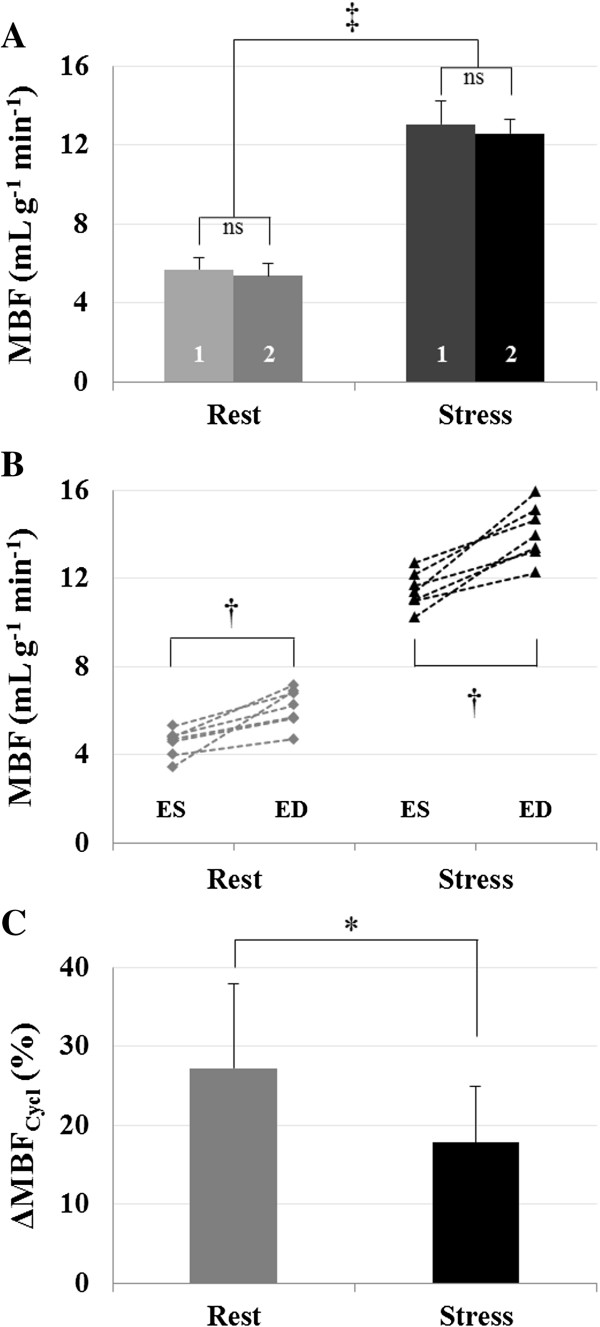
**Experimental results and statistics. A)** Mean MBF obtained within the group at rest (5.5 ± 0.6 mL g^-1^ min^-1^) and during adenosine stress (12.8 ± 0.7 mL g^-1^ min^-1^, *P*<0.0001, ‡). No statistical differences were found between the two observers (labels #1 and #2 on the bar plot). **B)** Individual minimum and maximum MBF values found at ES and ED, respectively. Two-tailed paired *t*-test was performed to compare minimum and maximum MBF, respectively, at rest, 4.7 ± 0.8 mL g^-1^ min^-1^ and 6.5 ± 0.6 mL g^-1^ min^-1^ (*P* = 0.0007, †) and under stress, 11.7 ± 1.0 mL g^-1^ min^-1^ and 14.2 ± 0.7 mL g^-1^ min^-1^ (*P* = 0.0007, †). **C)** The mean relative variation ΔMBF_Cycl_ was found significantly different (27.2 ± 10.7% at rest and 17.8 ± 7.1% at stress (*P* = 0.014,*)).

For each temporal bin along the cardiac cycle, mean perfusion values were found in agreement between the two observers. No significant differences were found between the two observers, with a Pearson correlation coefficient of r = 0.88 at rest and r = 0.89 during stress. We found a mean MBF over the cardiac cycle of 5.5 ± 0.6 mL g^-1^ min^-1^ at rest (observer 1, 5.7 ± 0.6 mL g^-1^ min^-1^ and observer 2, 5.4 ± 0.6 mL g^-1^ min^-1^, *P* = 0.12) which was significantly different from 12.8 ± 0.7 mL g^-1^ min^-1^ obtained during stress (observer 1, 13.1 ± 1.2 mL g^-1 ^min^-1^ and observer 2, 12.6 ± 0.7 mL g^-1^ min^-1^, *P* = 0.38). Results are presented in Figure 5A. A significant difference in myocardial perfusion was found between rest and stress conditions (*P*<0.0001), leading to a mean perfusion reserve of 2.4 ± 0.2 within the group.

The two lowest values and the two highest values obtained within the cardiac cycle were identified and averaged for each studied animal. The minimum and maximum MBF values found at ES and ED were defined as ${\text{MBF}}_{\text{Min}}^{\text{Rest}/\text{Stress}}$ and ${\mathrm{MBF}}_{\mathrm{Max}}^{\text{Rest}/\text{Stress}}$ respectively. Individual results are reported in Figure 5B. Resting transmural MBF extrema were significantly different with ${\mathrm{MBF}}_{\text{Min}}^{\text{Rest}}$ = 4.7 ± 0.8 mL g^-1^ min^-1^ and ${\mathrm{MBF}}_{\mathrm{Max}}^{\mathrm{Rest}}$ = 6.5 ± 0.6 mL g^-1^ min^-1^ (*P* = 0.0007). During stress, they were also significantly different with ${\mathrm{MBF}}_{\mathrm{Min}}^{\mathrm{Stress}}$ = 11.7 ± 1.0 mL g^-1^ min^-1^ and ${\mathrm{MBF}}_{\text{Max}}^{\text{Stress}}$ = 14.2 ± 0.7 mL g^-1^ min^-1^ (*P* = 0.0007). The mean relative variation ΔMBF_Cycl_ within the group (Figure 5C), was found significantly different between both conditions. We found 27.2 ± 9.3% decrease from ED to ES at rest and 17.8 ± 7.1% during adenosine stress, with paired samples *t*-test *P* = 0.014. On the other hand, MPR was minimum at ED, 1.9 ± 0.1 and maximum at ES, 2.8 ± 0.3 (*P* < 0.0001). Individual quantifications as well as a statistic summary are reported in Table 2 for MBF and Table 3 for MPR.

**Table 2:** Individual myocardial blood flow obtained in 7 healthy rats at rest and during adenosine stress

**Rat**	**MBF rest (mL g**^**-1 **^**min**^**-1**^**)**				**MBF stress (mL g**^**-1 **^**min**^**-1**^**)**				**ΔMBF**_**Cycl **_**(%)**	
	**Min**	**Max**	**Mean**	**SD**	**Min**	**Max**	**Mean**	**SD**	**Rest**	**Stress**
1	5.5	7.3	6.3	0.6	12.7	13.7	13.2	0.7	25.2	7.3
2	5.4	6.6	5.9	0.5	11.9	13.7	12.7	0.7	19.1	12.8
3	3.5	6.8	4.8	1.0	10.5	14.8	12.3	1.4	48.5	29.2
4	5.3	7.3	6.2	0.6	12.3	15.0	13.5	1.0	28.2	17.8
5	4.5	6.1	5.3	0.5	12.8	15.0	13.7	0.8	26.4	14.7
6	4.0	5.7	5.1	0.6	10.6	13.8	12.5	1.0	29.5	22.8
7	4.7	6.3	5.1	0.5	10.8	13.5	11.8	0.9	25.3	19.8
Mean	4.7	6.6^†^	5.5	0.6	11.7	14.2^†^	12.8^‡^	0.9	27.2	17.8^*^
SD	0.7	0.6	0.6		1.0	0.7	0.7		9.3	7.1

Individual mean and standard deviation has been calculated over the entire cardiac cycle. Two-tailed paired *t*-test *P* values: ^*^P = 0.014 (ΔMBF_Cycl_), ^†^*P* = 0.0007 (maximum vs. minimum) and ^‡^*P*<0.0001 (stress vs. rest for Mean MBF).

**Table 3:** Individual myocardial perfusion reserve obtained in 7 healthy rats

**Rat**	**MPR**				
	**Min**	**Max**	**Mean**	**SD**	**ΔMPR (%)**
1	1.7	2.8	2.2	0.2	38
2	1.8	2.7	2.2	0.2	35
3	1.8	3.2	2.6	0.4	42
4	1.9	2.8	2.3	0.2	29
5	2.2	3.1	2.7	0.2	29
6	2.1	2.9	2.4	0.2	28
7	2.0	2.7	2.3	0.2	26
Mean	1.9	2.9*	2.4	0.2	32
SD	0.2	0.2	0.2		6

Two-tailed paired *t*-test *P<0.0001 (maximum vs. minimum).

### Relationship between heart rate and cyclic MBF variation amplitude

**Figure 6:**
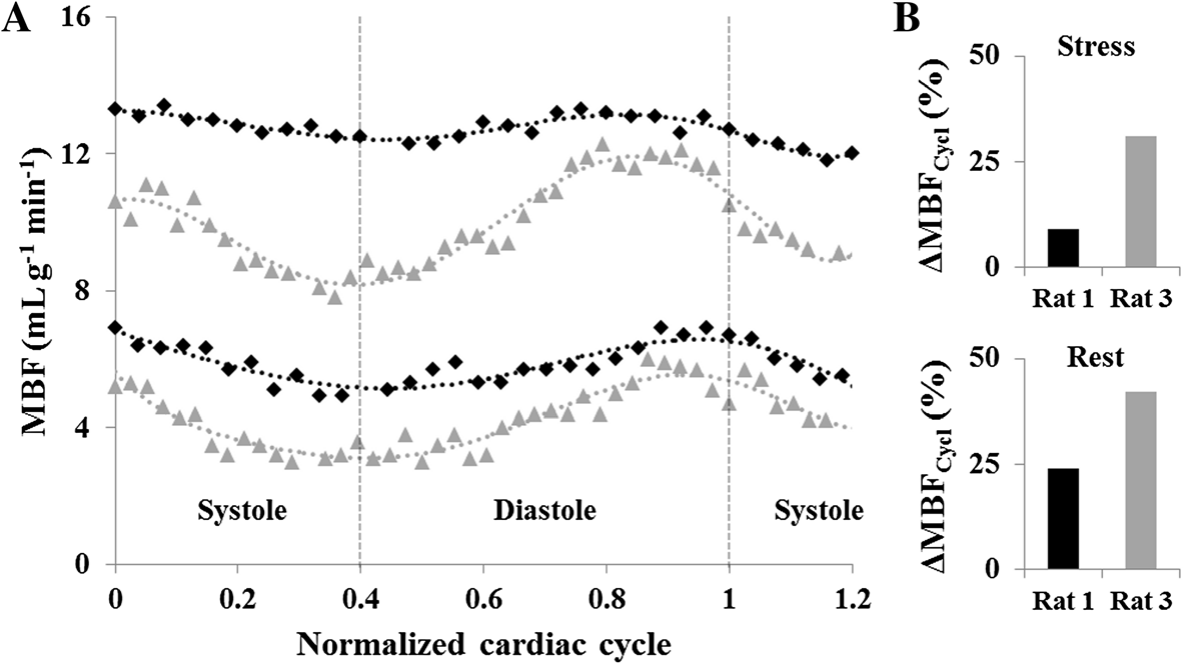
**Individual comparison of cyclic myocardial perfusion variation. A)** MBF is shown for two specific animals as a function of the cardiac cycle: Rat 1 with a heart rate of 370 bpm (black diamonds) and Rat 3 with a heart rate of 260 bpm (grey triangles). **B)** The variation ΔMBF_Cycl_ was higher for Rat 3 (42% at rest and 31% during stress) compared to Rat 1 (24% at rest and 9% during stress).

**Figure 7:**
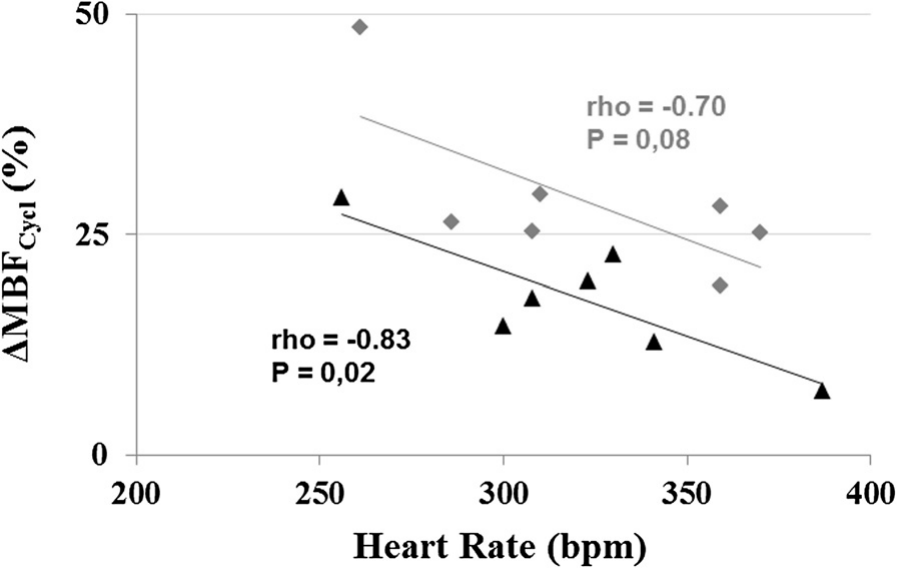
**Relationship between heart rate and amplitude of the relative MBF variation.** A linear group correlation exists between individual heart rate and the corresponding relative variation ΔMBF_Cycl_ during adenosine stress (black triangles, rho = −0.83, *P* = 0.02). Even though no correlation was found at rest (gray diamonds, rho = −0.70, *P* = 0.08), a strong dependence of ΔMBF_Cycl_ on heart rate can be seen.

In Figure 6A, the impact of the heart rate on the amplitude of MBF variation is highlighted. This graph shows the two extreme cases found within the group in terms of heart rate, i.e. the animal with the highest heart rate (Rat 1, 370 bpm) and the one with the lowest heart rate (Rat 3, 260 bpm). For clarity, a six orders polynomial regression was used to fit the data. ΔMBF_Cycl_ (Figure 6B) was lower for Rat 1 (24% at rest and 9% during stress) compared to the Rat 3 (42% at rest and 31% during stress). We verified whether a linear group correlation existed between individual heart rate and the corresponding relative variation ΔMBF_Cycl_ for both rest and stress conditions (Figure 7). There was a significant correlation between ΔMBF_Cycl_ and heart rate during stress (rho = −0.83, *P* = 0.02) but not at rest (rho = −0.67, *P* = 0.10).

## Discussion

In this study we report time-resolved absolute quantification of myocardial blood flow by CMR with a new approach based on a cine-FLASH labeling and readout module. The spatial resolution (313 × 625 μm^2^) and the sensitivity were sufficient to measure global myocardial perfusion and hemodynamic changes within the cardiac cycle in approximately 12 min. MBF maps were obtained with a high temporal resolution of 6 ms covering almost two cardiac cycles. The tagging scheme used here consists in rapidly repeated local inversions with precise timing within the cardiac cycle. It allows maintaining the labeled blood state throughout the experiment while a cine readout can be achieved during the remaining time with a steady-state acquisition. Tissue magnetization is thus driven into a perfusion-dependent equilibrium state so that signal changes that occur during the cardiac cycle can only reflect changes in momentary tissue blood flow. Arterial spin labeling measures the integrated effect of tagged blood flowing through the vasculature and remaining in the capillary system during the mean transit time (MTT). MTT is of the order of a second in the rodent heart and therefore much longer than a cardiac cycle. The averaged blood flow across the cardiac cycle reported here corresponds to the mean blood flow commonly defined by MBF = MBV/MTT which is understood on a much longer time scale than a cardiac cycle. By using high-temporal resolution cine readouts of 6 ms, signal that is acquired within a narrow window allows capturing the time-variant changes in the microvascular status of the myocardium, which is related to the number of patent myocardial microvessels. Thus, the variations observed at different time-points within the cardiac cycle have to be distinguished from the mean blood flow and are due to cyclic variations of momentary flow.

### Capillary flow dynamics

Figure 4 shows that cyclic temporal variations were measurable using the cine-ASL technique.

Left-coronary arterial blood flow is characterized by a diastolic-dominant inflow [35] whereas small distal venous flow exhibits a systolic-dominant pattern [36]. Our results are consistent with the expected physiological pattern of preferential coronary diastolic filling.

A clear and complete description of coronary flow patterns across the cardiac cycle in humans has been proposed by Davies et al. [34], who used intracoronary wires for measuring pressure and Doppler velocity waveforms. Microvascular MBF is closely related to macrovascular coronary flow such that these results can be confronted to ours. During systole, the authors observed a dominant forward-travelling pushing wave in proximal coronary arteries that is reflected when reaching the microvascular bed in distal coronary arterioles, causing blood to move in the opposite direction. Coronary arteries are also known to have a significant capacitance to accommodate for blood supply [37]. Also, during ventricular contraction, the compressive forces on the small vessels lying in the myocardium increase while venous outflow is in its dominant phase. One can therefore expect decreasing capillary flow during systole, which is consistent with our observations. During diastole, however, Davies and coworkers highlighted a dominant backward-travelling ‘suction’ wave initiated at the beginning of ventricular relaxation along with decreased compressive forces on capillaries and resistance of microvessels. Closure of the aortic valve in early diastole generates a late forward-travelling pushing wave which accelerates blood still further towards the myocardium. Most of the arterial inflow occurs during diastole with a velocity peak at early to mid-diastole. We observed the same pattern with a delay that corresponds to the time for the blood to reach the microvascular compartment.

We showed that momentary blood flow is non-uniform through the cardiac cycle and occurs mainly in diastole when myocardial tension is low. Therefore, the timing of data acquisition in perfusion CMR experiments may affect the measured MBF and might be considered in the evaluation of perfusion exams in both research and clinical practice. We note here that for studying temporal variations of perfusion using arterial spin labeling CMR, the underlying spin-exchange model has to be kept in mind. One-compartment models are valid for perfusion quantification in the heart [38], in which free diffusivity and complete exchange of the blood magnetization with the tissue during the presence of a spin in the arterial capillary compartment is assumed. However, the time scale relevant here is the delay between successive cardiac frames, which is much shorter than the time for spins to travel across the arterial capillary compartment, i.e. the time relevant for perfusion model validity. Taking into account spin exchange between perfusate and tissue, rapid MBF variations are observable if the exchange is fast on the scale of our frame rate. The significant cyclic perfusion change found in this study supports the assumption of sufficiently fast capillary/tissue spin exchange in myocardium. In the literature, there are few studies regarding changes in spin exchange rates in the context of cardiovascular pathologies. Since arterial spin labeling only uses water molecules, we can hypothesize that the fast exchange assumption should be preserved even across pathologies.

### Rest versus stress

Changes in MBF across the cardiac cycle were significant in this study with, in general, a similar behavior between rest and stress experiments. Nevertheless, two major differences arose from these results. First, maximum MBF appeared sooner within the cardiac cycle at stress than at rest. This was the case not only within the averaged group results, but also for each studied animal. This observation is also reflected in the MPR variations, in which a drop was found at the end of the cardiac cycle, corresponding to the delay between both physiological states. Second, we found a mean group decrease of MBF from ED to ES (ΔMBF_Cycl_) of 27.2 ± 9.3% at rest, which was significantly higher from ΔMBF_Cycl_ obtained during stress (17.8 ± 7.1%, *P* = 0.014).

In a study published by Kajiya et al. [37], the authors focused on the phase opposition of velocity waveforms between coronary arteries and veins. They introduced the unstressed volume (UV), which refers to a capacitance accommodating for blood supply during diastole without significant increase in UV pressure to propel venous outflow. But UV can become saturated when diastolic arterial inflow is higher than normal, such as in pharmacologic stress situations causing vasodilation. In this case, the pressure of ordinary capacitance exceeds the venous pressure causing diastolic venous outflow. Such theory might explain why the maximum MBF was found to appear sooner within the cardiac cycle during stress.

When administered intravenously, adenosine effect is mixed, endothelial dependent and non-dependent [39] and thus causes relaxation of smooth muscles as found inside the arterial walls. This action leads to vasodilation and increases arterial inflow along with a decreased resistance and an increased capacitance of microvessels. Here, the term vascular resistance refers to the impediment offered by the vascular bed to a unidirectional and constant blood flow. The concept of vascular impedance, however, involves the pressure-flow relations at each instantaneous timing change and refers to the impediment offered to flow at the input of a vascular bed where pulsatile flow is involved. Thus, vascular impedance takes into account not only the resistance but also dynamic effects and seems more appropriate in this context. It is defined as the ratio of arterial pressure and flow. By reaching the maximum coronary response using adenosine, microvessels are already subjected to very high blood flow but also to reduced arterial pressure [32], which in our experiments resulted in lower vascular impedance represented in our experiments by a lower ΔMBF_Cycl_ during stress.

### Relationship between heart rate and cyclic MBF variation amplitude

There was a linear correlation when comparing heart rate and ΔMBF_Cycl_ amplitude during stress (Figures 6 and 7). However, no significant correlation was found at rest which might be explained by the lower sensitivity of ASL at rest compared to stress. This might also be due to an insufficient number of animals to reach significance. There is a dependence of ΔMBF_Cycl_ on heart rate under stress, which could be present at rest as well. A likely explanation for this behavior is that the vascular system of the heart has a capacitive property when submitted to pressure variations at its entry. Analogies between electrical circuits and the vascular system, such as the Windkessel model, have been often used for modeling approaches [40]. In a similar way as electric current in a capacitor submitted to voltage variations, the pressure oscillations lead to flow oscillations whose amplitude depend on the frequency of the pressure variations, here given by the heart rate.

### Absolute MBF and MPR quantifications

It should be noted that all MBF values reported in this study, especially at rest, cannot be considered as physiological baseline values due to the influence of isoflurane anesthesia on coronary vasodilation [31,41]. Resting MBF within the group was 5.5 ± 0.6 mL g^-1^ min^-1^ and therefore similar to previously reported values in rats, which are in the range of 3.83−7.89 mL g^-1^ min^-1^, using CMR [18,23,42] or fluorescent microspheres [23,43,44]. MBF obtained during stress was 12.8 ± 0.7 mL g^-1^ min^-1^, and mean MPR was 2.4 ± 0.2. To our knowledge, MPR has not been reported to be more than 2.5 in rodents and our results were in accordance with earlier obtained values using CMR in comparison with fluorescent microspheres [23] or contrast-enhanced ultrasound [45,46]. However, MPR in this experiment was found higher than that reported by Croteau et al. (1.4 ± 0.5) in a study using positron emission tomography and ^13^N-NH_3_ as radiotracer [47]. In their article, the authors discussed the fact that vasodilation induced by the pharmacologic stress was not maximal in their experimental design with the commonly used clinical injection rate of 140 μg kg^-1^ min^-1^. In our first experimental trial, we also encountered difficulties to obtain a reproducible stress within a group of healthy rats using this typical infusion rate. Increasing twice the infusion rate and using a small injection line with inner diameter of 0.58 mm resulted in obtaining a maximal MPR more reliably.

### Study comparison and limitations

In humans, Radjenovic et al. [11] found no differences between systolic and diastolic MBF at rest and a significant MBF increase in diastole during adenosine-induced stress. The authors discussed that cyclic MBF changes may be present also at rest in humans but that the precision of the first-pass gadolinium-based MRI method used in their work might not be sufficient to assess them.

MBF quantification errors may be present by various sources such as respiratory motion. No respiratory gating was done with this sequence. Likely owing to the high number of signal averages performed with cine-ASL along with randomization of breathing events with myocardial contraction, impact of respiratory motion on MBF quantification and cyclic behavior is expected to be negligible. Although the heart lengthens longitudinally from systole to diastole, through-plane motion affects both control and tag scans in the same way so that their effects should cancel when calculating the difference maps. Effect of through-plane motion on the magnetization steady-state should also be relatively small considering the rather thick (2 mm) slices. With cine-ASL, temporal placement of the labeling pulse cannot be corrected once the acquisition started. Particular attention was paid to heart rate stability so as to ensure regular labeling efficiency throughout the entire 12 minutes scan. As shown in Table 1, the heart rate variation in this group of healthy rats did not exceed 15% at rest and 20% during stress, and one can therefore expect limited impact of label pulse timing imperfections on MBF quantifications. Heart rate variations can, however, be larger in models of cardiac pathology and would necessitate individual labeling efficiency determination.

Another potential source of error, especially when assessing dynamic perfusion changes over the cardiac cycle, are the short waiting periods before each ECG trigger that lead to interruptions of the longitudinal magnetization steady-state. Again, these interruptions affect tag and control scans in the same way, but in addition, the acquisition was extended over two cardiac cycles instead of one, such that the second cycle was not affected by the steady-state interruption. Figures 3 and 4 indeed prove good concordance between the dynamics of the initial phases of the first and the second observed cycle. Finally, it cannot be excluded that tissue and/or blood *T*_1_ as well as *T*_2_^*^ are submitted to changes throughout the cardiac cycle. *T*_1_ might in addition be altered in a pathological context when tissue structural changes play a role. As a limitation common to many ASL techniques, cine-ASL would indeed require the acquisition of a *T*_1_ map to be fully quantitative. In terms of cyclic changes, the influence of *T*_1_ variations across the cardiac cycle can, however, be expected to remain small and would only linearly affect the resulting MBF values. Given the short echo time used in the cine-ASL sequence, the influence of *T*_2_^*^ on the signal is also expected to remain small.

## Conclusion

To our knowledge, this study presents the first attempt to quantify and monitor cyclic MBF variation in vivo in rats using a new and fully non-invasive arterial spin labeling CMR protocol. These experiments were carried out in a group of healthy rats with a focus on cyclic changes at rest and during adenosine-induced stress. Our observations clearly show that, in rodents, blood flow is phase-dependent and differs significantly between systole and diastole, for both rest and stress conditions. We discussed rat myocardial perfusion changes in response to adenosine as an infused vasodilator in detail and provided complementary information on how cyclic perfusion changes are affected by the vasodilator. Also, maximum absolute blood flow occurs earlier under stress conditions than under resting conditions. This might become relevant when calculating perfusion reserve based on two measurements with equal trigger delays. Considering the nature of coronary hemodynamics, cyclic MBF changes may reveal new physiologic information because they are a function of coronary flow, myocardial contraction and microvascular condition. This technique may be useful in a pathologic context to study microvascular defects in non-ischemic heart disease models.

## Abbreviations

MBV: Myocardial blood volume; MBF: Myocardial blood flow; MTT: Mean transit time; ASL: Arterial spin labeling; CMR: Cardiovascular magnetic resonance; ES: End-systole; ED: End-diastole; ECG: Electrocardiogram; FLASH: Fast low-angle shot; TR: Repetition time; MPR: Myocardial perfusion reserve; ΔMBFCycl: Relative variation of myocardial blood flow across the cardiac cycle.

## Competing interests

TT was a recipient of a PhD fellowship “CIFRE” from Siemens Healthcare, France.

## Authors’ contributions

TT carried out the sequence design, conceived the protocol, performed the acquisition and analysis of data, and drafted the manuscript. TC created the theoretical model for perfusion quantification and participated in the revision of the manuscript. MB helped in the interpretation of data. FK participated in the study design, managed coordination and participated in the revision of the manuscript. All authors read and approved the final manuscript.
